# Dietary phytochemical index and psychological disorders in a large sample of Iranian adults: a population-based study

**DOI:** 10.1186/s41043-023-00456-5

**Published:** 2023-11-10

**Authors:** Zahra Darabi, Aazam Ahmadi Vasmehjani, Amin Salehi-Abargouei, Masoud Mirzaei, Mahdieh Hosseinzadeh

**Affiliations:** 1 Research Center for Food Hygiene and Safety, School of Public Health, Shahid Sadughi University of Medical Sciences, Yazd, Islamic Republic of Iran; 2 Yazd Cardiovascular Research Center, Shahid Sadoughi University of Medical Sciences, Yazd, Islamic Republic of Iran

**Keywords:** Psychological, Diet, Nutrition, Food

## Abstract

**Background:**

Intake of plant foods and phytochemicals can decrease the odds of mental health.

**Aim:**

The aim of study is to investigate the association between Dietary Phytochemical Index (DPI) with symptoms of depression, anxiety and stress in a large sample of Iranian adults.

**Methods:**

In this cross-sectional study, a total of 7385 adults aged 20–50 years old were provided from the recruitment phase of Yazd Health Study, a population-based cohort research on Iranian adults. Food intakes were assessed via a validated and reliable food frequency questionnaire. Symptoms of depression, anxiety and stress were assessed using a validated Depression, Anxiety and Stress Scales questionnaire with 21-items. DPI was calculated by the percent of daily energy intake taken from phytochemical-rich foods.

**Results:**

After adjustment for potential confounders, normal weight subjects in the highest tertile of DPI had lower odds of depression (OR 0.65; 95% CI 0.44–0.97) and anxiety symptoms (OR 0.65; 95% CI 0.45–0.93) compared with those in the lowest tertile. However, the apparent association was not found between depression, anxiety and stress in overweight and obese subjects.

**Conclusion:**

We found a significant association between DPI and mental health in normal weight adults. Prospective studies are required to approve these conclusions.

## Background

The most common psychiatric disorders, depression and anxiety are among the comprehensive health problems [1]. Results of previous studies reported the prevalence of major depressive disorder and anxiety was 4.7% and 7.3% in the worldwide, respectively [1, 2]. The prevalence of psychiatric disorders was 10.81% in IRAN. It is reported that around 7 million of the Iranian population endure from one or more of the psychiatric disorders [3]. Since psychiatric disorders force a powerful economic burden and help to impair the quality of life, it is critical to find a suitable approach for prevention and treatment of these disorders [4].

Previous research has shown that diet is significantly associated with psychiatric disorders [5, 6]. Results of cross-sectional study showed that high intake of healthy foods such as vegetables and fruits and low intake of unhealthy foods such as soda, French fries, fast food, sweetened fruit drinks, cake, cookies, pie, ice cream and frozen desserts inversely associated with odds of psychological distress [5]. Another study has shown that a higher intake of fruit and vegetable reduced the risk of depression and also higher intake of added sugars increased the risk of anxiety in males [6].

Food groups such as fruits, vegetables, whole grains, nuts, legumes and essential oils are rich in phytochemicals that have antioxidant and anti-inflammatory properties [7, 8]. Phytochemicals are natural, non-nutritive bioactive compounds. Examples of phytochemicals are phenolic compounds, isoprenoids and organosulfur compounds [4]. The main potential effects of phytochemicals are anti-estrogenic, immunomodulatory, cardioprotective, anticarcinogenic, anti-inflammatory and antioxidant activities [9–11]. Based on the health booster effect of phytochemicals, McCarty proposed a dietary phytochemical index (DPI), which was characterized according to the percent of daily energy intake taken from phytochemical-rich foods. DPI could be a good indicator of diet quality and may be useful for clinical purposes [12].

Several studies have been evaluated the association between DPI and health status [13, 14]. One study has shown that higher score of DPI is associated with a lower risk of abdominal obesity and hypertriglyceridemia [15]. Another study reported that participants in the highest quartiles of DPI had lower the risk of hyperinsulinemia after 3-years of follow-ups compared to participants in the lowest quartiles of DPI [16]. Cross-sectional study was conducted in IRAN, and this study has reported that women in the highest tertile of DPI had a lower prevalence of depressive symptoms, anxiety and psychological distress compared with those in the lowest tertile [4]. Another cross sectional reported that higher score of DPI is associated with lower odds of depression in adolescents [17]. Noruzi et al. reported that high adherence to DPI can decrease the odds of depression in overweight and obese women [18].

Results of previous study have shown that inflammation and oxidative stress have role playing in the pathophysiology of psychiatric disorders [19, 20]. Due to the anti-inflammatory and antioxidant effects [8], it has been suggested this index is mental-protective. Few studies investigated the relationship between dietary phytochemical index and psychological disorders, and the aim of study is to investigate the association between DPI and symptoms of depression, anxiety and stress among a large sample of Iranian adults.

## Method

### Study design and participants

This cross-sectional study was performed on the framework of Yazd Health Study (YaHS) and Taghzieh Mardom-e-Yazd (TAMYZ). Detailed information about the study design, sampling procedures, participants' characteristics and data collection process has been previously published [21]. Briefly, YaHS was a prospective cohort that carry out 10,000 participants from the adult population aged 20–70 years old inhabit in Yazd city during 2014–2016. The participants were involved 50 people from 200 clusters (5 males and 5 females from each age10 years group) that were randomly chosen from the Yazd community based on residential postal codes. TAMEZ is an ongoing population-based cross-sectional study that assessed the dietary food and supplement intake of people living in Yazd city. All 10,000 participants of YaHS are included in TAMEZ. In this study, we used data from 7385 adults (49.7% male and 50.3% female) whose both psychological profile and dietary intake information had been completed at the time of analysis.

### Dietary assessment

Dietary assessment is done via validated 178 item food frequency questionnaire (FFQ) which modified version of a previously validated 168-item that 10 questions particularize consumption of Yazd-specific food items was added to the original 168-item FFQ [21, 22]. Participants were asked that reported frequency and usual amount intake of item food then converted to grams using guidelines of household scales [23].

### Phytochemical index calculation

The DPI was computed based on the method developed by McCarty in 2004; {DPI = [daily energy derived from phytochemical-rich foods (kcal)/total daily energy intake (kcal)] × 100} [12]. Fruits, vegetables, legumes, whole grains, nuts, soy products, seeds and olive oil were considered as phytochemical-rich foods. Potatoes were not included as vegetables because of their low phytochemical content. Natural fruit and vegetable juices as well as tomato sauces were included in the fruit and vegetable groups because these are also considered as their high phytochemicals content [24].

### Assessment of psychological profile

The psychological condition of the participants was assessed via the validated Depression, Anxiety and Stress Scales (DASS-21) questionnaire [25]. Each part of DASS-21 (depression, anxiety, psychological distress) consists of 7 questions. The response was divided into: zero, low, medium, and high with a score between 0 and 3. DASS-21 is the shorten DASS-42. So, the total points for each item should be duplicated by 2. Therefore, depression, anxiety and stress are characterized by the following scores: ≥ 10, ≥ 8 and ≥ 15.

### Anthropometric assessments

Body weight was measured with the minimum possible clothes and barefoot standing in the middle of Omron BF511 (Japan) portable digital. Height was determined to the nearest 0.1 cm in a standing position using a stadiometer with shoulders in a relaxed position and arms hanging freely and without shoes. Body mass index (BMI) (kg/m^2^) was calculated through the following formula: weight (kg) divided by height squared (m^2^).

### Physical activity assessment

Physical activity was determined by International Physical Activity Questionnaire (IPAQ). The short form of IPAQ questionnaire was used to assess frequency and time spent on sedentary, moderate, intensity activities, according to a list of common activities of daily life, over the past week. Physical activity levels were expressed as the metabolic equivalent of hours per week [26].

### Assessment of other variables

Data about age, gender, marital status, education level, job and history of chronic disease were collected via a self-administered questionnaire.

### Statistical analysis

General characteristics across tertile of DPI were expressed as means ± SDs and numbers and percentages for continuous and categorical variables, respectively. ANOVA and chi-square test were used for examining the differences across tertiles of DPI for continuous and categorical variables, respectively. Binary logistic regression has been used in crude and multivariable-adjusted models to determine ORs and 95% CIs for psychological profiles across tertiles of DPI. First, we controlled for age (continuous), sex (categorical) and energy intake (kcal/d) then for marital status (married, single and widowed or divorced), physical activity (sedentary/moderate/active), intake of supplement (never, 1–3 in mount, 2–4 in week, 5–6 in the week, one a day, two a day and more than two a day), history of chronic disease (yes/no, including of hypertension, diabetes, cardiovascular disease, cancer and dyslipidemia) and special diet (yes, no). Stratified analyses by BMI status (overweight and obese > 24.9 and normal weight ≤ 24.9 kg/m^2^) were also done in crude and adjusted models. P trend was determined by considering tertiles of DPI as ordinal variables in the logistic regression analysis. All statistical analyses were performed using SPSS version 23. *P* < 0.05 was designed to be statistically significant.

## Results

### Characteristics of participants

The psychological status of the participants is reported in Table 1. The prevalence of depression, anxiety and stress were, respectively, 8.1%, 10.5% and 3.3% in the study population. The general, demographic and anthropometric characteristics of the study population across tertiles of DPI are presented in Table 2. No significant difference was seen in marital status, physical activity, intake of supplement, employment status and education across tertiles of DPI but age of participants, history of chronic disease and special diet were significant between tertiles. Weight and BMI are significantly greater in the highest tertile of DPI compare to the lowest tertile. Food groups and nutrient intake of study population across tertile categories of DPI are reported in Table 3. Subjects in the highest tertile of DPI compared to those in the lowest tertile had lower intakes of vitamin E, vitamin B2, vitamin B12, vitamin D, total fat, monounsaturated fatty acids (MUFA), saturated fatty acids (SFA), and higher intake of iron, magnesium, vitamin A, vitamin B1, folic acid, biotin, pantothenic acid and vitamin C. Also, subjects in higher tertile of DPI had significantly lower energy intake compared to subjects in lowest tertile.

**Table 1:** The prevalence of depression, anxiety and stress across tertiles of DPI

DPI tertiles					
Variable	Total	T1	T2	T3	*P* value*
*Depression [n (%)]*					
Yes	578 (8.1)	208 (8.7)	179 (7.5)	191 (8)	0.33
No	6591 (91.9)	2189 (88.4)	2199 (87.1)	2203 (92)	
*Anxiety [n (%)]*					
Yes	754 (10.5)	277 (11.6)	228 (9.6)	249 (10.4)	0.08
No	6415 (10.5)	2120 (88.4)	2150 (90.4)	2145 (89.6)	
*Stress [n (%)]*					
Yes	238 (3.3)	87 (3.6)	74 (3.5)	77 (3.2)	0.57
No	6931 (96.7)	2310 (96.4)	2304 (96.9)	2317 (96.8)	

*DPI* Dietary phytochemical index

*Obtained from χ2 test

**Table 2:** General and anthropometric characteristics of participants across the tertiles DPI

DPI tertiles									
Variable	Total		T1		T2		T3		*P* value*
	Mean	SD	Mean	SD	Mean	SD	Mean	SD	
Weight (kg)	72.74	5.09	72.05	14.84	72.63	14.49	73.54	14.61	0.01 >
BMI (kg/m^2^)	27.00	5.09	26.81	5.21	26.84	4.98	27.35	5.06	0.01 >
MET_min/week	901.16	905.16	908.59	898.30	895.66	887.64	899.16	929.33	0.87
Age [*n* (%)]									0.02
20–29 years	1584 (21.5)		565 (22.9)		533 (21.7)		488 (19.81)		
30–39 years	1598 (21.6)		572 (23.2)		506 (20.6)		520 (21.1)		
40–49 years	1586 (21.5)		541 (21.9)		551 (22.5)		494 (920)		
50–59 years	1402 (19)		488 (19.3 (		467 (19)		447 (18.1)		
60–69 years	1215 (16.4)		431 (17.5)		395 (15.6)		389 (15.8)		
Marriage [*n* (%)]									0.49
Single	856 (11.6)		258 (11.3)		301 (11.9)		270 (10.7)		
Married	6264 (85.1)		2096 (83)		2072 (82.1)		2096 (83)		
Widowed or divorced	240 (3.3)		76 (3)		75 (3)		89 (3.05)		
Smoking [*n* (%)]									0.41
Never smoker	113 (1.6)		2107 (83.5)		2103 (83.3)		2118 (83.9)		
Current smoker	6238 (78.8)		271 (10.7)		260 (10.3)		235 (9.3)		
Ex_smoker	766 (10.6)		37 (1.5)		33 (1.3)		43 (1.7)		
Multi-vitamin supplement [*n* (%)]									0.05
Never	6.407 (86.1)		2122 (85.4)		2159 (88)		2126 (85)		
1–3 mount	482 (6.5)		187 (7.5)		132 (5.4)		163 (6.5)		
Once a week	171 (2.3)		55 (2.2)		54 (2.2)		62 (2.5)		
2–4/week	36 (0.5)		13 (0.5)		10 (0.4)		13 (0.5)		
5–6/week	38 (0.5)		13 (0.5)		9 (0.4)		16 (0.6)		
Once a day	209 (2.8)		57 (2.3)		64 (2.6)		88 (3.5)		
Two a day	87 (1.2)		35 (1.4)		24 (1)		28 (1.1)		
More than two a day	8 (0.1)		2 (0.1)		2 (0.1)		4 (0.2)		
Gender [*n* (%)]									0.53
Men	3672 (49.7)		1212 (49.1)		1242 (50.6)		1219 (49.5)		
Women	3711 (50.3)		1258 (50.9)		12,119 (49.4)		1242 (50.4)		
Job [*n* (%)]									0.72
Unemployed	1415 (19.5)		462 (19)		496 (20.6)		457 (19)		
Government employee	3537 (48.8)		1183 (48.7)		1169 (48.5)		1185 (49.20)		
Manual worker	247 (3.4)		90 (3.7)		79 (3.3)		78 (3.2)		
Freelance job	2046 (28.2)		693 (28.5)		665 (27.6)		688 (28.6)		
Education [*n* (%)]									0.10
Illiterate	1776 (24.1)		610 (24.8)		593 (24.3)		573 (23.3)		
Middle school	2095 (28.5)		717 (29.2)		708 (29)		670 (27.2)		
Associate’s degree	2289 (31.1)		761 (30.9)		739 (30.3)		789 (32.1)		
Bachelor’s degree	995 (13.5)		319 (13)		332 (13.6)		344 (14)		
Master’s degree and doctor	205 (2.8)		52 (2.1)		68 (2.8)		85 (3.5)		
Special diet [*n* (%)]									0.01 >
Yes	1081 (17.30)		415 (24.48)		280 (13.92)		386 (18.93)		
No	5164 (82.69)		1280 (75.51)		1731 (86.07)		1653 (81.06)		
History of chronic disease [*n* (%)]									0.01 >
Yes	4290 (56.6)		1042 (41.3)		1085 (43)		1157 (45.8)		
No	3284 (43.4)		14.82 (58.7)		1440 (57)		1368 (54.2)		

*DPI* Dietary phytochemical index, *BMI* Body mass index

*Obtained from ANOVA for continuous variables and χ^2^ test for categorical variables

**Table 3:** Multivariable-adjusted dietary intakes across the tertiles of DPI (mean values and standard deviation)

DPI tertiles									
Variable	Total		T1		T2		T3		*p* value*
	Mean	SD	Mean	SD	Mean	SD	Mean	SD	
Energy intake (kcal)	2897.00	1378.62	3102.68	1463.82	2651.40	1284.49	2937.00	1344.04	0.001 >
Protein (g)	114.07	60.19	119.12	66.9	106.27	53.91	116.84	58.27	0.001 >
Carbohydrate (g)	406.92	216.65	423.01	240.59	371.94	196.19	425.82	206.49	0.001 >
Fat (g)	111.95	72.85	129.01	83.68	101.79	64.69	105.05	65.56	0.001 >
Energy intake of fruits	222.67	197.52	144.54	92.82	207.76	136.71	315.68	273.45	0.001 >
Energy intake of vegetables	72.24	106.40	54.95	46.84	66.00	67.04	95.75	162.45	0.001 >
Energy intake of legumes	54.37	71.71	42.46	40.22	51.30	51.06	69.35	104.07	0.001 >
Energy intake of nuts	148.78	239.27	112.36	154.00	127.60	157.00	206.36	343.99	0.001 >
Energy intake of whole grain	189.34	269.93	105.00	87.49	141.15	118.32	321.83	412.28	0.001 >
SFA (g)	31.04	18.36	36.09	22.23	28.62	15.58	28.42	15.38	0.001 >
PUFA (g)	28.79	24.46	30.17	25.32	26.13	21.83	30.08	25.83	0.001 >
MUFA (g)	34.23	24.03	40.29	28.8	30.89	20.70	31.5	20.48	0.001 >
Cholesterol (mg)	394.88	391.32	452.29	526.38	368.05	300.01	364.34	295.73	0.001 >
EPA (mg)	20	70	20	90	20	70	20	70	0.36
DHA (mg)	70	20	70	230	60	180	70	20	0.41
Vitamin A (RAE/day)	23.77	33.59	18.34	29.60	20.53	24.14	32.44	42.55	0.001 >
Vitamin E (mg/day)	11.36	11.66	11.87	12.29	11.17	11.09	11.04	11.55	0.001 >
Vitamin D (µg)	1.48	2.10	1.61	2.77	1.45	1.85	1.37	1.46	0.001 >
Vitamin K	169.74	249.23	165.95	261.94	163.41	233.46	179.85	251.27	0.04
Vitamin C (mg)	210.59	188.73	158.57	106.22	196.09	141.71	277.09	261.10	0.001 >
VitaminB1 (mg)	2.23	1.03	2.19	1.06	2.08	0.09	2.42	1.09	0.001 >
VitaminB2 (mg/)	2.37	1.22	2.50	1.33	2.24	1.16	2.39	1.14	0.001 >
VitaminB3 (mg/)	28.48	15.45	29.65	17.44	26.27	13.75	29.53	14.69	0.001 >
Biotin (mg/day)	22.53	18.04	22.39	19.68	21.42	16.09	23.77	18.10	0.001 >
Pantothenic acid (mg/day)	6.60	3.75	6.10	2.98	6.12	2.98	7.57	4.79	0.001 >
VitaminB6 (mg)	2.55	1.64	2.61	1.90	2.38	1.44	2.66	1.52	0.001 >
Folic acid (µg)	379.87	228.00	345.92	190.03	354.02	205.30	439.65	268.94	0.001 >
VitaminB12 (µg)	6.07	6.32	6.76	6.93	5.76	6.37	5.69	5.52	0.001 >
Calcium (mg)	967.97	489.50	983.09	536.04	921.24	470.69	999.59	454.69	0.001 >
Magnesium (mg)	339.33	179.66	319.98	162.10	315.67	166.02	382.32	200.58	0.001 >
Zinc (mg)	11.94	6.03	12.26	6.52	11.07	5.28	12.50	6.14	0.001 >
Fe (mg)	43.19	82.30	38.55	94.63	38.47	40.63	52.55	97.93	0.001 >

*DPI* Dietary phytochemical index, *SFA* Saturated fatty acid, *PUFA* Polyunsaturated fatty acid, *MUFA* Monounsaturated fatty acid, *RAE* Retinol activity equivalents

*Obtained from ANOVA

### Psychological status and DPI

Mental health across tertiles of DPI for the participant in crude and adjusted OR (95% CIs) stratified by BMI are shown in Table 4. In subjects with normal weight (BMI ≤ 24.9), a significant protective association was found between DPI and odds of depression (OR 0.82; 95% CI 0.68–0.99; *p *= 0.04) and anxiety (OR 0.83; 95% CI 0.71–0.98; *p *= 0.03) in crude model. Also, there was a significant decreasing trend in the odds of anxiety and depression across increasing tertiles of the DPI (*P* trend: 0.03) and (*P* trend: 0.04), respectively. This association remained significant after adjusting for potential confounders including age, energy intake, sex, marital status, physical activity, supplement use, history of chronic disease, smoking and special diet (OR 0.79; 95% CI 0.64–0.97; *p* = 0.02) and anxiety (OR 0.79; 95% CI 0.66–0.95; *p *= 0.01). Also, there was a significant decreasing trend in the odds of anxiety and depression across increasing tertiles of the DPI (*P* trend = 0.02 and 0.01, respectively). There was no significant association between stress and tertiles of DPI in crude and adjusted models. However, in overweight and obese subjects, no significant association between tertiles of DPI and psychologic disorders was observed both in crude and adjusted model.

**Table 4:** Odds ratio depression, anxiety and stress across tertiles of DPI (multivariable-adjusted odds ratios and 95% confidence intervals)

Variable	DPI tertile			*P* trend‡
	T1	T2	T3	
*BMI* ≤ *24.9*				
Depression				
Crude	1.00	0.71 (0.50–1.01)	0.69 (0.48–1.00)	0.04
Adjusted model1*	1.00	0.67 (0.47–0.96)	0.69 (0.47–0.99)	0.03
Adjusted model2**	1.00	0.58 (0.39–0.86)	0.65 (0.44–0.97)	0.02
Anxiety				
Crude	1.00	0.75 (0.55–1.03)	0.43 (0.51–0.98)	0.03
Adjusted model1*	1.00	0.72 (0.52–0.99)	0.69 (0.5–0.96)	0.02
Adjusted model2**	1.00	0.65 (0.46–0.93)	0.65 (0.45–0.93)	0.01
Stress				
Crude	1.00	0.83 (0.49–1.4)	0.81 (0.47–1.41)	0.90
Adjusted model1*	1.00	0.78 (0.45–1.33)	0.81 (0.47–1.40)	0.43
Adjusted model2**	1.00	0.54 (0.29–0.99)	0.74 (0.61–1.22)	0.28
*BMI* > *24.9*				
Depression				
Crude	1.00	0.94 (0.72–1.22)	1.03 (0.80–1.32)	0.80
Adjusted model*	1.00	0.71 (0.50–1.01)	0.69 (0.48–1.00)	0.78
Adjusted model2**	1.00	1.02 (0.77–1.35)	1.08 (0.82–1.42)	0.57
Anxiety				
Crude	1.00	0.84 (0.67–1.06)	0.97 (0.78–1.28)	0.82
Adjusted model1*	1.00	0.84 (0.67–1.07)	0.97 (0.78–1.21)	0.99
Adjusted model2**	1.00	0.89 (0.69–1.14)	0.98 (0.77–1.24)	0.86
Stress				
Crude	1.00	0.86 (0.58–1.28)	0.91 (0.62–1.33)	0.95
Adjusted model1*		0.86 (0.52–1.29)	0.92 (0.62–1.39)	0.70
Adjusted model2**		0.94 (0.61–1.65)	0.99 (0.65–1.51)	0.99

*DPI* Dietary phytochemical index

*Adjusted for age, gender and total energy intake

**Adjusted for model 1 and marital status, physical activity, supplement use, history of chronic disease, smoking and special diet

^†^These values are odds ratios (95% CIs)

^‡^Obtained from logistic regression by considering tertiles of DPI as ordinal variable

## Discussion

In the current study evaluating the relation between DPI and psychological disorders, we observed that higher intake of phytochemical-rich foods was inversely associated with odds of depression and anxiety symptoms in participant with normal BMI. These protective relations remained significant after adjustment for a wide range of possible confounding variables. To the best of our knowledge, this study is the first study that explores the relationship between DPI and psychological disorders in a large population from a Middle-Eastern country.

Long-standing studies have shown that food or dietary pattern with fluent phytochemicals is protective for mental health; for instance, results of a cohort study show that higher intake of flavonols, flavones and flavanones was inversely associated with risk of depression [27]. Another study reported that higher intake of phenolic acid, flavanones and anthocyanins significantly decreases risk of depressive [28]. Some studies reported that “prudent,” “lacto-vegetarian” and “healthy” dietary patterns characterized by high intakes of phytochemical-rich foods including fruits, vegetables, medium fat dairy, nuts, legumes and fish were protectively associated with depression and anxiety [29, 30]. In addition, some other studies reported the health benefit effects of DPI on abdominal obesity, lipid profile and insulin resistance [15, 16]. In contrast with our results, a cohort study reported that high intakes of flavan-3-ols and anthocyanins were not significantly associated with depression risk in women [27]. Another study explained that higher consumption of high phytochemical foods such as fruits and vegetables was not associated with depressive symptoms among males [31] Also, there was not significant association between DPI and inflammatory markers as a mediator of psychological disorders in another research [32].

Inflammatory markers such as serum levels of C-reactive protein, tumor necrosis factor alpha and interleukin 6 were related to mental health [33, 34]. So, the pro-inflammatory cytokines have role playing in numerous clinical distinct of depression such as disrupted serotonin metabolism, overactivity of the hypothalamus–pituitary–adrenal axis and neurodegenerative symptoms [35]. Phytochemicals are disrupt the production of pro-inflammatory mediators such as those derived from the arachidonic acid cascade. These derived components have inflammatory effects via modulating neuro-inflammation by cooperation with p38 signaling cascades and STAT-1 [36]. Fruits and vegetables are the main sources of folate. Results of some studies have shown that there is association between serum folate and dietary intake of this vitamin and depression [29, 37, 38]. It can affect on mental health via methylation in the nervous system [37]. Numerous psychiatric disorders were associated with oxidative stress. Brain is susceptible to oxidative damage. The brain consumes high oxygen and it produces higher free radicals. Brain has lipid-rich structure that is vulnerable to oxidation [39, 40]. Phytochemicals are powerful antioxidants. It can able to neutralize free radicals by donating an electron or hydrogen atom [8].

Our results have shown no significant association between stress and DPI. Few studies investigated the association between food groups and symptom stress. Results of cohort study showed that the Mediterranean Diet Score was inversely associated with psychological distress. This score was characterized by high intake of vegetables, fruit, cereals, legumes and fish [41]. The results of our studies were inconsistent with previous studies. The reason for inconsistency was probably using different questionnaires to diagnose psychological distress. Also, this study was conducted on a large sample size and both men and women.

There was no significant association between DPI and psychological disorders in overweight and obese subjects. In consistent with our result, one study reported that there was no association between traditional and lacto-vegetarian dietary patterns with high phytochemical food such as vegetables, fruit and wholegrain and psychological disorders in obese participant [30]. BMI was considered as independent predictor of mental disorders [42], which obesity and overweight mitigate the phytochemical effects on mental disorders. Results of experimental study have shown that rats with 50% weight gain in 8 months compared to controls presented depression and anxiety-like behaviors [43]. Obesity often comes with a low-grade chronic inflammatory state that systemic markers of inflammation were increased [44]. As mentioned above, inflammation has a role in the pathogenesis of mental disorders.

On other hand, obesity and overweight usually have negative body image. There is evidence that shows relationship between depressive symptoms and anxiety with body image dissatisfaction [45]. Some studies revealed that obesity acts a role in the development of psychopathological disorders such as anxiety and psychosis via beging low self-esteem [46, 47].

This study has several strengths. We adjusted for a wide range of potential confounders that might affect psychological conditions, also the large represented sample size of the study including both sexes. Stratified results based on BMI as an important dietary variable related to psychological disorders were presented. This study has some limitations. First, the cross-sectional study was not shown causal relationship between mental health and DPI. Second, we undertake to control for manifest confounding factors and, however, may have been some residual confounding. Third, DPI does not include non-caloric phytochemical-rich food such as green and black tea which are sources of phytochemicals. Finally, DASS questionnaire does not provide diagnostic of disorders and only estimates symptoms of depression and anxiety.

## Conclusion

We understand that higher score of DPI including higher intake of fruits, vegetables, nuts, olive and olive oil, legumes and whole grains was correlated with lower odds of depressive symptoms and anxiety in subjects with normal BMI, but not in overweight subjects. These findings are needed to be examined with prospective studies to verify the causality between DPI and mental health.

## Data Availability

Available upon request.

## Abbreviations

DPI: Dietary phytochemical index
DASS: Depression, Anxiety and Stress Scales questionnaires
YaHS: Yazd health study
TAMYZ: Taghzieh Mardom-e-Yazd food
BMI: Body mass index
FFQ: Frequency questionnaire
IPAQ: International Physical Activity Questionnaire
MET: Metabolic equivalent
MUFA: Monounsaturated fatty acids
SFA: Saturated fatty acids
